# Giant Circular Dichroism in Chiral Broad Flat‐Band Quasi‐BIC Metasurface

**DOI:** 10.1002/nap2.70106

**Published:** 2026-04-25

**Authors:** Chunhao Xu, Jietao Liu, Guofeng Song

**Affiliations:** ^1^ Institute of Semiconductors Chinese Academy of Sciences Beijing China; ^2^ College of Materials Science and Opto‐Electronic Technology University of Chinese Academy of Sciences Beijing China; ^3^ Institute of Intelligent Photonics Nankai University Tianjin China

**Keywords:** bound state in the continuum, chirality, flat‐band, metasurface

## Abstract

Compact, high‐efficiency chiral light sources are essential for on‐chip integrated photonics, yet simultaneously achieving giant circular dichroism (CD), ultrahigh quality factor (Q), and angular robustness remains unresolved. Here, we report a chiral flat‐band metasurface that unifies giant CD, ultrahigh Q, and full angular resilience by merging symmetry‐broken bound states in the continuum (BICs) with a dispersion‐engineered photonic band. Platform‐tilted double rectangular nanopillars break both in‐plane and out‐of‐plane mirror symmetries, generating an intrinsical chirality and an ultraflat photonic band. The resulting quasi‐BIC resonance features a high Q‐value ≈ 1969 and near‐unity transmission CD (> 0.99). Crucially, the dispersionless ultraflat band ensures stable resonance and maintains high CD across a moderate angular range of ± 4°. Furthermore, strong slow light field confinement effectively quenches lateral leakage, enabling high ellipticity (*ρ* > 0.99) and *Q* ≈ 1108 even in finite‐sized structures of merely eight periods. By synergistic integrating giant chiral response with flat‐band photonics, this strategy establishes a scalable route to ultralow threshold–integrated chiral photonic‐crystal surface‐emitting lasers (PCSELs) and angle robust chiral sensors. Compared with existing chiral flat‐band structures, our design achieves substantial, concurrent enhancement of circular dichroism and a quality factor, establishing a compelling platform for integrated chiral photonic devices.

## Graphical abstract

Tuning the near‐field coupling between adjacent nanopillars gradually flattens the photonic band. Reducing the interpillar spacing strengthens mode hybridization, yielding an ultraflat dispersion that hosts a high‐Q quasi‐BIC. This work delineates the physical mechanism linking structural parameters to flat‐band formation, enabling giant circular dichroism and robust angular performance in chiral metasurfaces.

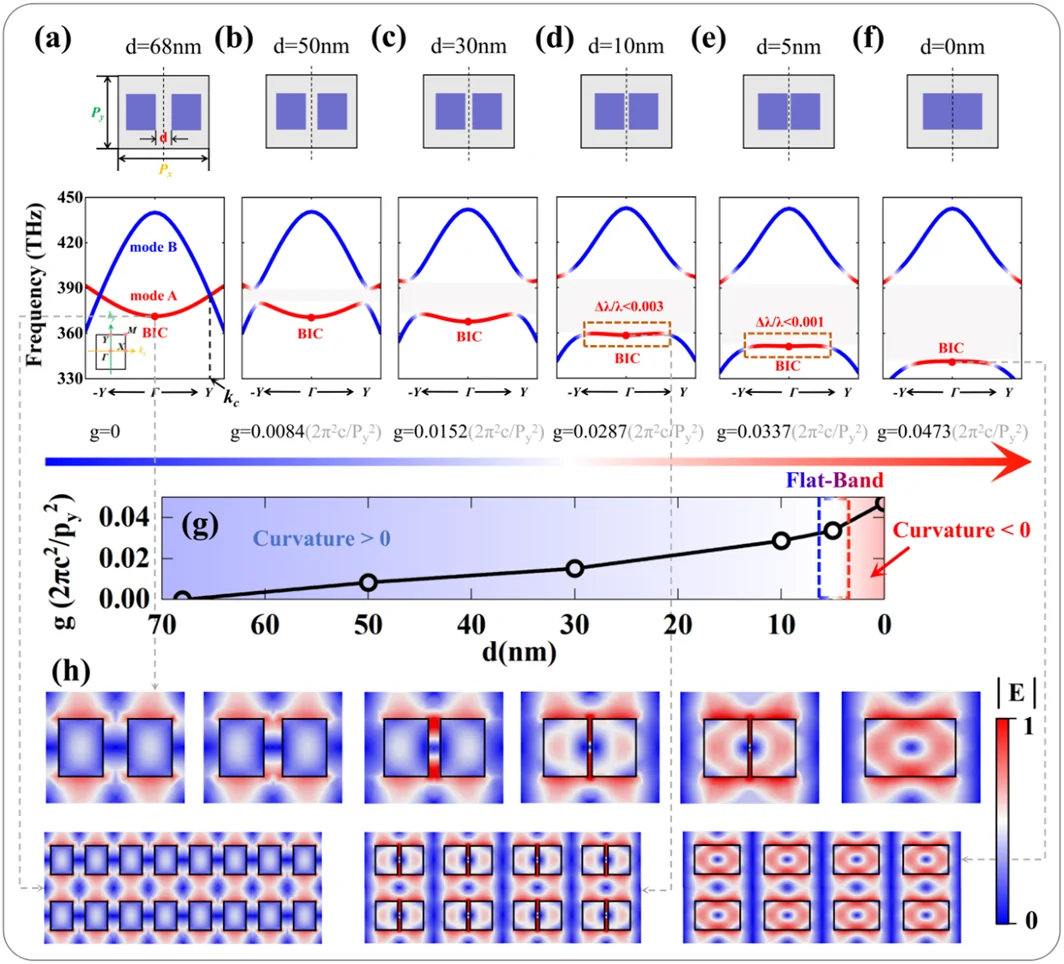

## 1 Introduction

Chirality originates from the breaking of mirror symmetry or rotational symmetry, underpinning critical applications across diverse technological fields. Optical chirality, the differential interaction with circularly polarized light (photon spin angular momentum), is quantified as per circular dichroism (CD) as the difference in absorption, reflection, or transmission of right versus left circularly polarized (RCP/LCP) light [1]. Conventional optical chirality from bulk materials or chiral molecules suffers from intrinsically weak responses [2]. Metasurfaces with subwavelength architectures harness resonant light–matter interactions, offer a compact and efficient alternative [3, 4, 5, 6, 7], and enable strong differential reflection or transmission responses under RCP and LCP illumination [8]. Meanwhile, high‐Q resonances have been realized via bound states in the continuum (BICs) [9, 10, 11, 12]. BICs represent special eigenstates that remain localized within the radiation continuum with a theoretically infinite lifetime; they can be converted into quasi‐BICs (q‐BICs) by breaking the in‐plane inversion symmetry of the structure and introducing perturbations. Leveraging this property, several structures supporting high‐Q circularly polarized emission have been successfully demonstrated [13, 14, 15, 16, 17]. However, chirality in most such metasurfaces originates from either oblique incidence or structural anisotropy. Tilted nanostructures have been utilized to break both in‐plane and out‐of‐plane symmetry, thereby inducing strong near‐field chirality, which has experimentally yielded high CD and high Q‐factors [18, 19, 20]. Such chiral metasurfaces hold broad application prospects in chiral light sources and detectors, chiral sensing, valleytronics, and asymmetric photocatalysis [18, 21, 22]. Nevertheless, none have achieved chirality‐stable resonances with frequency invariance over a wide range of incident angles. Moreover, advancing integration demands ever more compact architectures. Recently, besides BICs, flat bands offer an alternative strategy to enhance light–matter interactions [23, 24, 25, 26, 27, 28]. Their weak dispersion or even dispersionless energy bands yields low group velocities and high density of states (DOS), enabling slow light–enhanced in‐plane field confinement—suppressing lateral leakage and boosting coupling between photons and gain media. However, integrating flat bands into chiral structures has proven exceptionally challenging. A recent demonstration based on patterned meta‐atoms validated the concept [29], yet its circular dichroism (< 0.6) and Q (< 400) remain orders of magnitude below practical requirements.

Simultaneously, significant progress has been made in achieving near‐unity circular dichroism using quasi‐BIC resonances in the nonlinear regime. For instance, asymmetric silicon metasurfaces have been shown to generate strong chiral responses at the third harmonic frequency, leveraging the enhanced local fields of high‐Q resonances [30]. Although these nonlinear platforms offer unique opportunities for frequency conversion and active chiral photonics, their linear optical response—particularly the simultaneous achievement of ultrahigh Q‐factor, angular robustness, and giant circular dichroism—remains less explored. Moreover, the performance of such nonlinear chiral metasurfaces is often intrinsically tied to the pump intensity and nonlinear conversion efficiency, which limits their direct application in compact, low threshold–integrated light sources.

Here, we demonstrate a chiral flat‐band metasurface based on symmetry‐protected BICs engineered into q‐BICs to achieve giant CD and ultrahigh Q. By tuning intermodal coupling, we collapse the photonic band into an ultraflat profile, while simultaneously, tilted double rectangular nanopillars break both in‐plane and out‐of‐plane symmetry to yield near‐unity CD (> 0.99). Compared with existing designs, our structure maintains a comparable angular range of incidence while achieving higher CD. Such photonic flat bands offer significant advantages, as their characteristic slow light effect provides excellent in‐plane field confinement, suppressing lateral leakage and radiative losses induced by size effects. These attributes establish our design as an ideal platform for highly compact, integrated, and circularly polarized light sources, particularly for ultralow‐threshold, highly integrated chiral PCSELs.

## 2 Results

By judiciously engineering metasurface lattice parameters, flat‐band dispersion supporting high‐quality factor (high‐Q) resonances can be realized [23, 24, 25]. Figure 1a illustrates the proposed tilted nanopillar structure and its corresponding photonic band diagram. The lattice periods are set as *P*_*x*_ = 392 nm and *P*_*y*_ = 318 nm. The finite element method (FEM), with COMSOL Multiphysics, is used for the investigations. The metasurface consists of an array of double rectangular GaAs nanopillars on a glass substrate, capped with a silicon oxide layer to maintain mirror symmetry in the vertical direction and to avoid influence from additional diffraction channels. In the simulation, the refractive indices of GaAs and glass are set as 3.55 and 1.46, respectively, and material dispersion is not considered. The nanopillars of height *t* = 300 nm are tilted by the angle *θ* toward the *y*‐axis.

**FIGURE 1:**
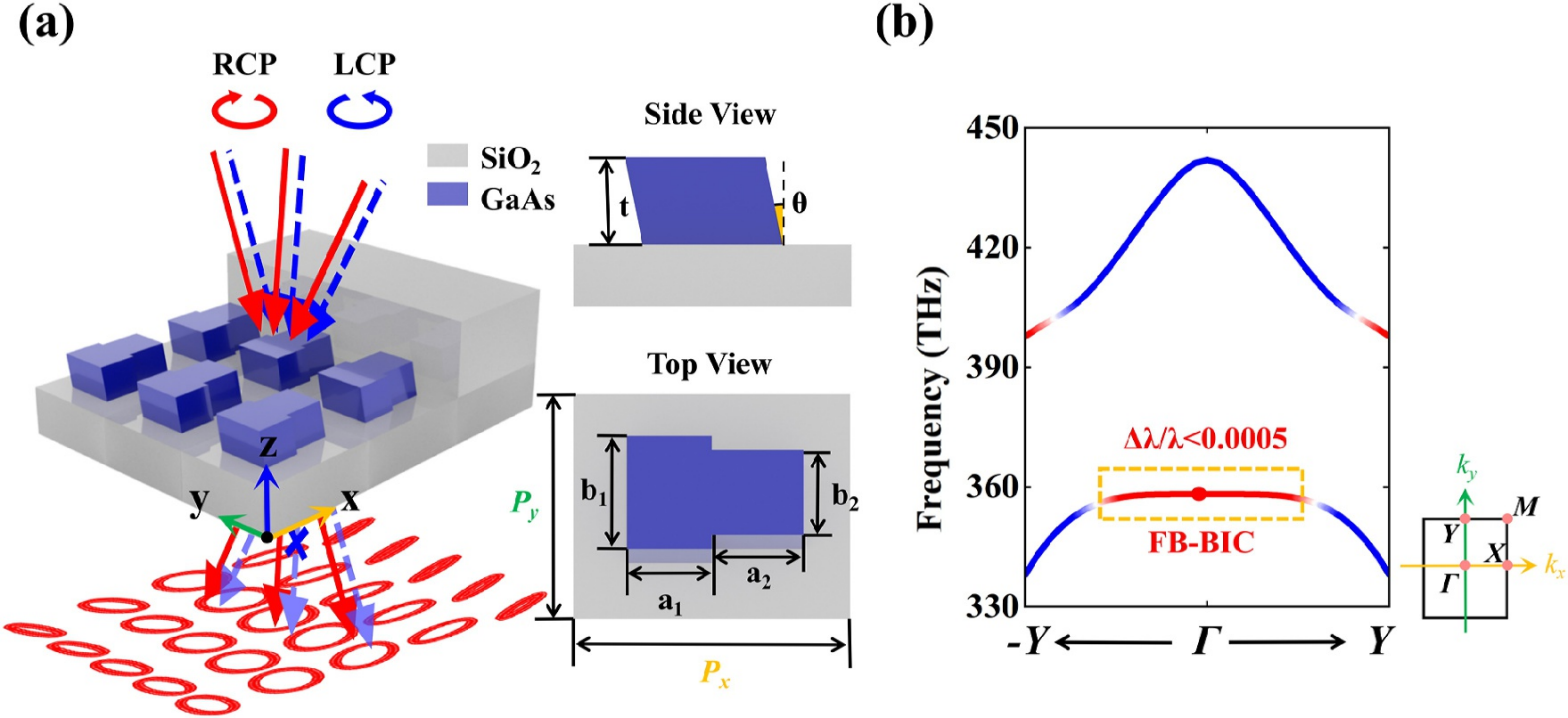
The schematic of the engineering chiral metasurface. (a) One unit cell of the designed metasurface—the tilted dual‐rectangular nanopillar structure. (b) Photonic band diagram of the proposed metasurface. FB‐BIC with a broad flat band is predicted.

From the band analysis result in Figure 1b, one can see that the proposed structure offers a broad flat‐band along the *Γ*–*Y* direction in the *k*‐space, with a relative bandwidth of Δλ/*λ* < 0.0005. This mode exhibits a flat‐band BIC (FB‐BIC) at the *Γ* point, possessing a high‐Q. The flat‐band dispersion characteristic significantly surpasses most reported weakly dispersive chiral structures, laying a physical foundation for the subsequent realization of angle‐independent chiral responses. To elaborate on the mechanism behind the formation of flat‐band dispersion, we present the geometric configurations of photonic crystals with different separations, *d*, and their corresponding band diagrams for transverse electric (TE) modes, and the electric field distribution of the BIC are shown in Figure 2. The structural parameters of the nanopillars are fixed as *a*_*1*_ = 128 nm and *b*_*1*_ = 164 nm. As the separation *d* between adjacent nanopillars decreases, the coupling between the two modes intensifies and becomes increasingly pronounced. The curvature of the target band near the *Γ* point is gradually reduced due to this coupling, eventually approaching a flat band. In the temporal coupled‐mode theory, altering the spacing between adjacent nanopillars doubles the period and halves the Brillouin zone. Consequently, the guided mode band originally located at the edge of the Brillouin zone is folded into the *Γ–Y* direction, forming two coupled modes. The coupling strength in this direction can be expressed as

(1) $$U\approx g\cdot \left|k_{y}\right|$$

where g is the near‐field coupling coefficient, whose magnitude is primarily controlled by the nanopillar spacing *d*. As shown in Figure 2, the near‐field coupling coefficient *g* gradually increases from 0 as the spacing *d* decreases. This process, which arises from the simultaneous breaking of vertical and in‐plane symmetries, is termed symmetry‐breaking coupling [31]. The Hamiltonian of this coupling mechanism can be written as

(2) $$H=\left(\begin{array}{cc} \omega_{1} & U\\ U & \omega_{2} \end{array}\right)+i\left(\begin{array}{cc} \gamma_{1} & \Gamma e^{-i\phi }\\ \Gamma e^{i\phi } & \gamma_{2} \end{array}\right)$$

where *ω*_*1,2*_ and *γ*_*1,2*_ are the real and imaginary parts of the two uncoupled modes, respectively. The antidiagonal term *Γ* corresponds to the coupling rate between the two modes via the radiative channels. The phase shift *ϕ* is the relative dephasing when they radiate into the continuum. To elucidate in a simplified and fundamental manner how mode coupling induces variation in the band structure (real‑part dispersion), we consider a lossless ideal system. The eigenvalues of the Hamiltonian for this system are calculated as follows:

(3) $$\omega_{\pm }=\frac{\omega_{1}+\omega_{2}}{2}\pm \sqrt{{\left(\frac{\omega_{1}-\omega_{2}}{2}\right)}^{2}+U^{2}}$$

**FIGURE 2:**
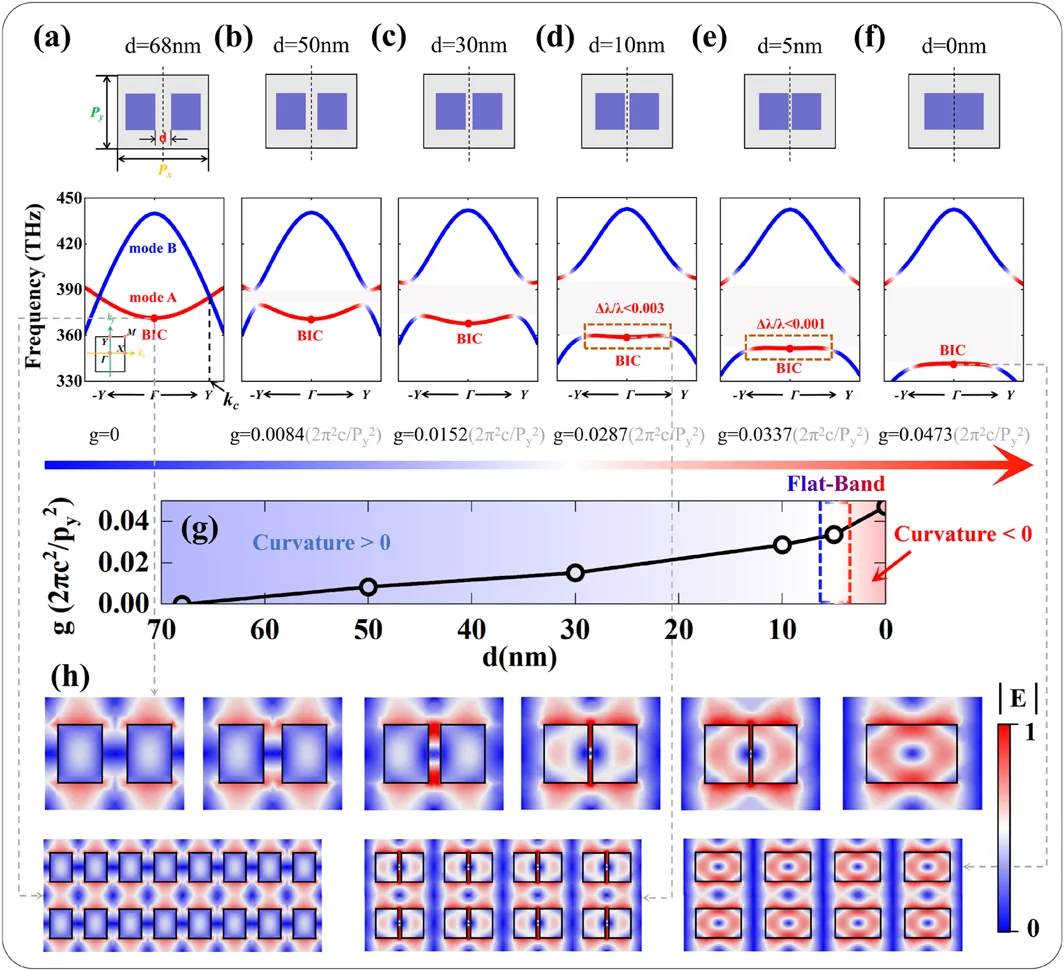
(a–f) Geometric structure of the metasurface, corresponding evolution of photonic band diagrams, and electric field distribution (h) at the BIC resonance for inter‐center spacings *d* of 68 nm, 50 nm, 30 nm, 10 nm, 5 nm, and 0 nm between the two adjacent rectangular nanopillars. (g) The trend and calculated near‐field coupling coefficient, *g*, derived from band structure fitting using Equation (3).

Figure 2a–f illustrates the evolution of the band structures for different nanopillar separations, *d*, revealing distinct frequency variations of Mode A and Mode B. Figure 2a shows the uncoupled case (*d* = 68 nm), where Mode A and Mode B remain independent and evolve along their respective original dispersion curves—the frequency of Mode A increases slowly with the wavevector *k*_*y*_, exhibiting a small positive dispersion with positive curvature at the *Γ* point, whereas Mode B changes more rapidly. The two modes cross near *k*_*y*_ = *k*_*c*_. Under weak coupling conditions (*d* = 50 nm), the coupling strength *U* is small, and weak coupling occurs only near *k*_*c*_, resulting in a noticeable avoided crossing, as shown in Figure 2b. As the separation *d* decreases, the near‐field coupling coefficient *g* gradually increases (Figure 2g), driving the system into a strong coupling regime. With increasing *k*_*y*_, *U(k*_*y*_ *) ≫ (ω*_*1*_ *(k*_*y*_ *) – ω*_*2*_ *(k*_*y*_ *))/2*, causing the coupling term *U* to dominate the expression under the square root over a broader wavevector range. At this point, according to Equation (3), the frequencies of the upper and lower modes can be approximated as follows:

(4) $$\omega_{\pm }=\frac{\omega_{1}+\omega_{2}}{2}\pm U$$

In Figure 2c and d, for *d* = 30 and 10 nm, respectively, it can be observed that the curvature of the lower mode band gradually decreases with increasing coupling strength. Mode A is notably suppressed near the *Γ* point, and its frequency at *k*_*y*_ = 0 decreases as *d* decreases. This is primarily because the coupling strength between the two modes at the *Γ* point is not simply approximated by Equation (1); instead, it exhibits a nonzero effective coupling strength that increases as *d* decreases. When *g* becomes sufficiently strong (Figure 2e), corresponding to a separation of 5 nm, the curvature of the lower mode band reaches exactly zero, satisfying the condition for a flat band. Band fitting yields a coupling coefficient of *g* = 0.0337 (2π^2^c/P_y_^2^) at this point, with Δλ/*λ* < 0.001 within the wavevector range of ± 0.1 × (2π/P_y_). As shown in Figure 2f, when the two rectangular nanopillars merge, the curvature of the lower mode undergoes a reversal, resulting in negative curvature. Figure 2h illustrates the evolution of the electric field distribution at the BIC resonance frequency as the center‐to‐center spacing, d, between two adjacent rectangular nanopillars gradually decreases. When the spacing is larger (*d* = 68 nm), the electric field modes of the two nanopillars are largely independent, exhibiting strong localization but weak mutual interaction. As d decreases, the near‐field coupling between the nanopillars progressively strengthens, and the electric field distribution transitions from independent modes to a strongly coupled superposition state, revealing distinct mode hybridization. When the spacing is further reduced to *d* = 5 nm, the electric field becomes highly localized within the gap between the nanopillars and forms a symmetric distribution, which corresponds to the formation condition of a flat band in the band structure. This evolution of the electric field directly elucidates the physical mechanism by which the near‐field coupling coefficient g increases with decreasing interpillar distance. It is this progressively enhanced coupling that drives strong interaction between the two original modes, ultimately leading to band flattening near the *Γ* point and the formation of a high‐Q flat‐band BIC resonance.

To transform this structure into a chiral configuration, we maintained the center‐to‐center spacing between the nanopillars to ensure the stability of the flat‐band structure although modifying the shape parameters of the right‐hand nanopillar, as shown in Figure 1a. Considering that altering the parameters of the right rectangular nanopillar would enhance symmetry‐breaking coupling, we selected a spacing of *d* = 10 nm—which does not yet yield a perfectly flat band—as the baseline for further adjustment. As shown in Figure 3, this rectangular nanopillar structure possesses mirror symmetry along both the *x*‐ and *y*‐directions. Mode A decouples from free space at the *Γ* point, representing a typical symmetry‐protected BICs. The Q factor of this BIC is approximately 1.87 × 10^9^. The adjusted parameters are *a*_*2*_ = 148 nm and *b*_*2*_ = 130 nm. Additionally, the rectangular nanopillars were intentionally tilted to completely break the structural symmetry. Specifically, the nanopillars were tilted along the *y*‐direction with an inclination angle *θ* = 3.7°. The asymmetric deformation applied to one of the nanopillars eliminates the high‐aspect ratio gaps between them, thereby improving fabrication feasibility. As illustrated in Figure 1b, compared to the pre‐modified structure, this asymmetric deformation enhances the coupling effect between Mode A and Mode B, making the curvature of the eigenfrequency of Mode A near the *Γ* point closer to zero, resulting in Δλ/*λ* < 0.0005. The flat band corresponds to a wavelength of approximately 837 nm. Our chiral design introduces two types of perturbations: an out‐of‐plane structural tilt angle *θ* and an in‐plane asymmetric deformation. The latter can be quantified by the parameter *Δb = b*_*1*_ *–b*_*2*_. Together, these two perturbations break all mirror symmetries resulting in an impact of both perturbations on the quality factor as illustrated in Figure 3. It can be observed that compared to applying only a single perturbation, the combination of both does not lead to an order‐of‐magnitude difference in the quality factor.

**FIGURE 3:**
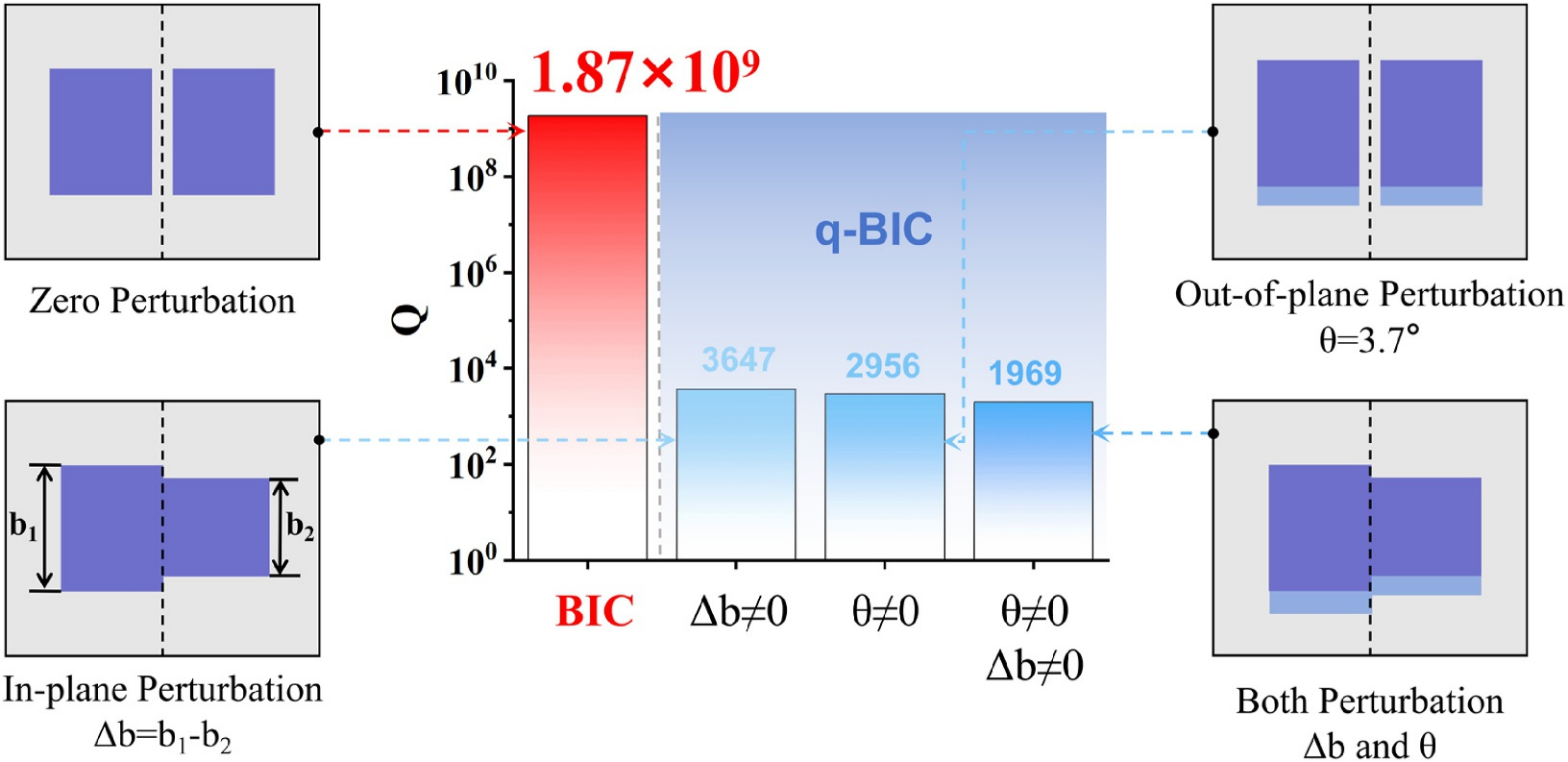
Influences of in‐plane perturbation *Δb* and out‐of‐plane perturbation *θ* on the Q factor. The deformation parameter is defined as *Δb = b*_*1*_ *–b*_*2*_.

To elucidate the influence of the tilted structure on the polarization of radiation, we provide a detailed analysis of how various parameters of the chiral configuration affect the polarization state of the emitted light. The phase difference *Δφ* between the *x*‐ and *y*‐polarized electric field components is primarily influenced by the lattice parameters *b*_*1*_ and *b*_*2*_ and can be precisely tuned by adjusting a scaling factor *α* applied to these structural parameters. By setting the two structural parameters to *αb*_*1*_ and *αb*_*2*_, respectively, we performed structural simulations. Figure 4a specifically illustrates the effect of the scaling factor *α* on the phase difference *Δφ*. It is observed that *Δφ* undergoes significant variation with changes in *α*, yet it remains remarkably stable and unaffected by variations in the tilt angle *θ*. Notably, when *α* = 1, *Δφ* consistently remains at π/2, satisfying the condition for generating circular polarization. To achieve strong chirality, precise control over the phase difference is required. This decoupling arises from the distinct symmetry‐breaking pathways associated with each geometric parameter. The in‐plane asymmetry parameter Δ*b* (controlled via *α*) primarily modifies the local electromagnetic environment and the effective refractive index experienced by the two orthogonal polarization components, thereby directly determining their phase retardation. In contrast, the out‐of‐plane tilt angle *θ* predominantly influences the radiation strength (magnitude) of the x‐polarized component by disrupting the vertical mirror symmetry, while leaving the relative phase accumulation between the x‐ and y‐polarized channels largely unaffected. Consequently, *Δφ* remains invariant under variations of *θ*, enabling independent control of phase and amplitude—a feature that is essential for achieving precise circular polarization conditions without complex iterative adjustments. Furthermore, we calculated the distinct effects of the tilt angle on the *x*‐ and *y*‐polarized electric field components for different values of *b*_*2*_. For clearer comparison of radiation under varying conditions, the radiated electric fields were normalized by setting the *x*‐polarized component *E*_*x*_ at *b*_*2*_ = 140 nm and a tilt angle of 6° to unity. As shown in Figure 4b, when only an in‐plane perturbation is introduced (*θ* = 0°), the radiation is linearly polarized along the *y*‐direction. This radiation originates from the breaking of mirror symmetry in the *x*‐direction, which transforms the symmetry‐protected BIC into a quasi‐BIC. At this stage, constrained by the remaining mirror symmetry in the *y*‐direction, the radiated light couples only to *y*‐polarization, and its intensity is influenced by the degree of in‐plane perturbation *Δb*. A decrease in *b*_*2*_ increases *Δb*, leading to a reduction in the quality factor and a corresponding enhancement of the radiated electric field, which aligns with the established relationship between perturbation strength and the Q factor in BICs. The quality factor, representing the capability to confine light, is defined in eigenmode analysis, as the ratio of the real part to the imaginary part of the mode frequency. Figure 4c details the relationship between the Q factor and the perturbation parameter *Δb*, confirming an inverse square proportionality. This relationship verifies that the high‐Q resonance in our structure originates from the distortion of a symmetry‐protected BIC. Returning to Figure 4b, for *θ* > 0°, the introduction of the out‐of‐plane perturbation (tilt angle *θ*) increases the radiated field *E*_*x*_ (solid lines) while keeping *E*_*y*_ (dashed line) unchanged, thereby meeting the condition for circular polarization. The independence of *E*_*y*_ from the tilt angle indicates its sole dependence on the in‐plane asymmetry parameter *Δb*. In contrast, *E*_*x*_ increases with the tilt angle at rates influenced by *Δb*. When the tilt angle reaches a specific value where *E*_*x*_ = *E*_*y*_, the condition for circularly polarized radiation is satisfied, yielding maximum chirality. Figure 4d presents the radiation ellipticity *ρ* under various conditions. The detailed calculation method of ellipticity is provided in Supporting Information S1. For different *b*_*2*_ values, the structure can achieve very high ellipticity at specific tilt angles. Specifically, for *b*_*2*_ = 130 nm and *θ* = 3.7°, the ellipticity *ρ* exceeds 0.99. The ellipticity achieved by our structure is comparable to the best results reported in previous studies on chiral configurations [19].

**FIGURE 4:**
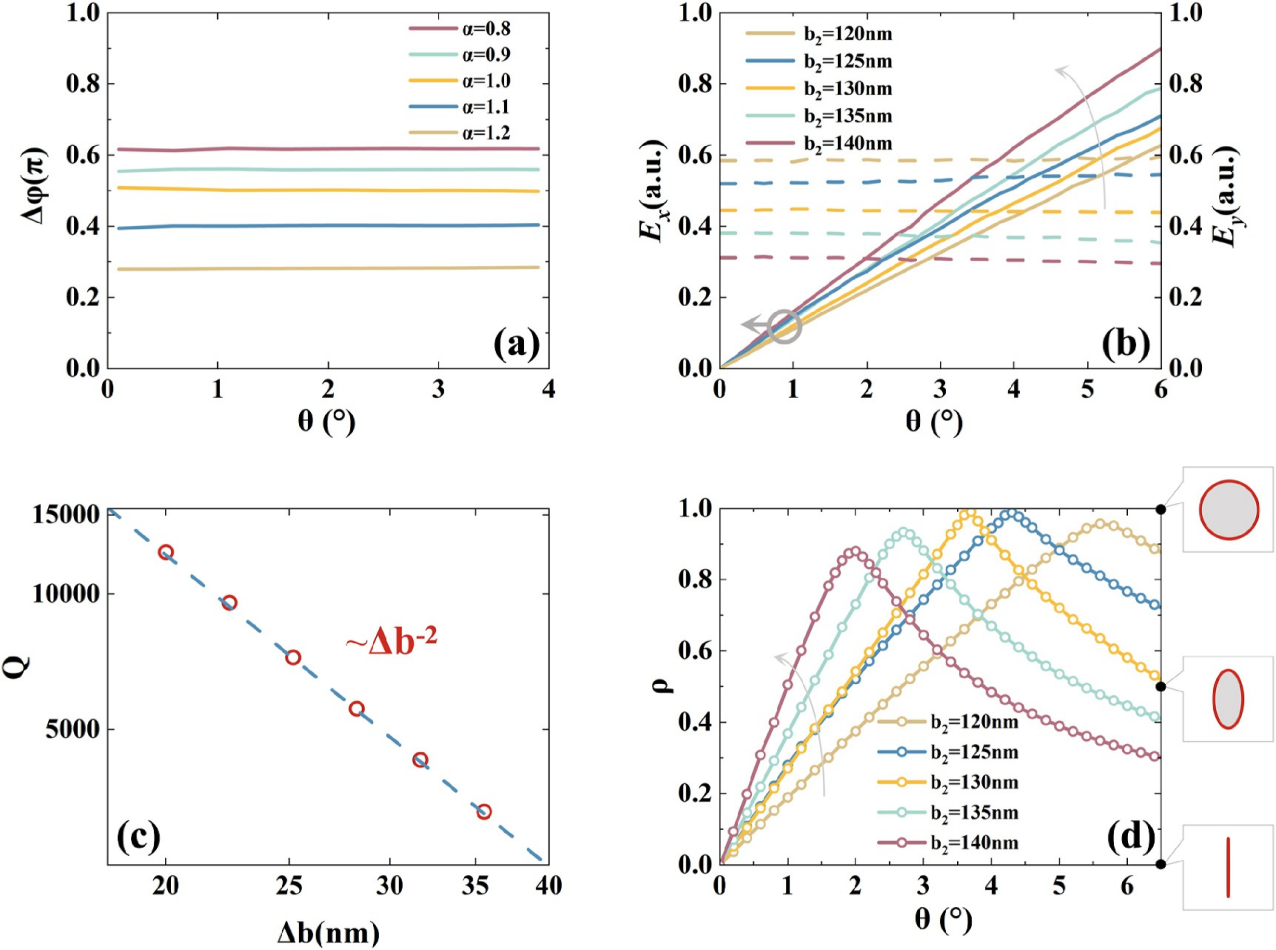
(a) Phase difference *Δφ* between the *xy* polarization components of the electric field versus the nanopillar tilt angle *θ* for different scaling factors *α* applied to Parameters *b*_*1*_ and *b*_*2*_. (b) Variation of the *xy* polarization components of the electric field with the nanopillar tilt angle *θ* under different degrees of in‐plane asymmetry. (c) Quality factor as a function of the asymmetry factor *Δb*; the dashed line represents a fit based on an inverse‐square relationship. (d) Ellipticity *ρ* versus the nanopillar tilt angle *θ* for varied *b*_*2*_.

We simulated the transmission spectra when the ellipticity *ρ* approaches unity and investigated the influence of thickness on transmittance, observing a strong resonance near 837.2 nm accompanied by pronounced CD. The proposed structure exhibits anisotropy, which inevitably gives rise to nonzero cross‐circular transmission coefficients *T*_*LR*_ and *T*_*RL*_, where *T*_*LR*_ denotes the LCP transmittance under RCP illumination and *T*_*RL*_ represents the RCP transmittance under LCP illumination. The structural anisotropy introduces a phase difference between the transmitted *x*‐ and *y*‐polarized light, which generates nonzero *T*_*LR*_ and *T*_*RL*_. In our design, this effect significantly reduces the achievable transmission circular dichroism. Because the phase shift induced by anisotropy depends on the thickness, we simulated *T*_*LR*_ at 837 nm for different structural thicknesses *t* to mitigate its impact on the transmission CD. As shown in the Figure 5a, *T*_*LR*_ varies linearly with the thickness *t*, reaching a value close to zero at *t* = 300 nm. In Figure 5b, we present the transmission spectrum and the corresponding transmission CD for *t* = 300 nm. The definition and the calculation method of CD are provided in Supporting Information S1. It can be seen that by optimizing the thickness parameter, we achieved nearly zero *T*_*LR*_ and *T*_*RL*_ while generating a sharp Fano resonance around 837 nm. At 837.2 nm, vanishing *T*_*LL*_ engenders giant circular dichroism exceeding 0.99.

**FIGURE 5:**
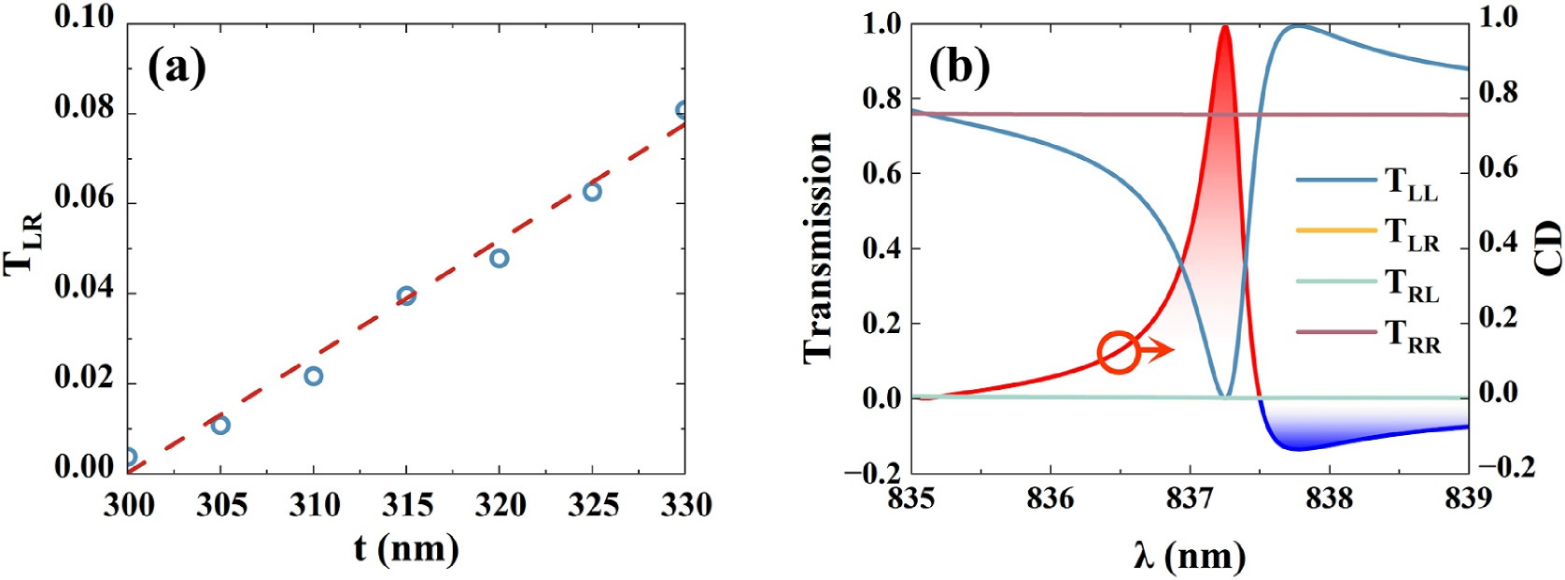
(a) Linear fit of the transmittance as a function of nanopillar thickness *t*. (b) Transmission spectrum and *CD*_*T*_ spectrum of the tilted nanopillar structure at *t* = 300 nm.

Benefiting from the excellent flat‐band dispersion, our structure exhibits a stable resonance frequency and high CD over a moderate angular range along the *y*‐direction [29, 31]. Figure 6a shows a structure under oblique incidence, where *β* is defined as the angle of incidence along the *y*‐direction. As shown in Figure 6b, we present the transmission circular dichroism across a broad range of incidence angles *β*. It can be observed that the proposed structure maintains remarkably high CD over a moderate angular range (± 4°). Figure 6c and d display the transmission spectra and the corresponding CD at incidence angles of 2° and 4°, respectively, revealing an extremely stable resonance wavelength. Compared to previous studies on flat‐band chiral structures, our design achieves a comparable angular operation range while establishing near‐unity transmission CD, a performance exceeding that of most conventional chiral metasurfaces relying on off‐normal excitation or structural anisotropy.

**FIGURE 6:**
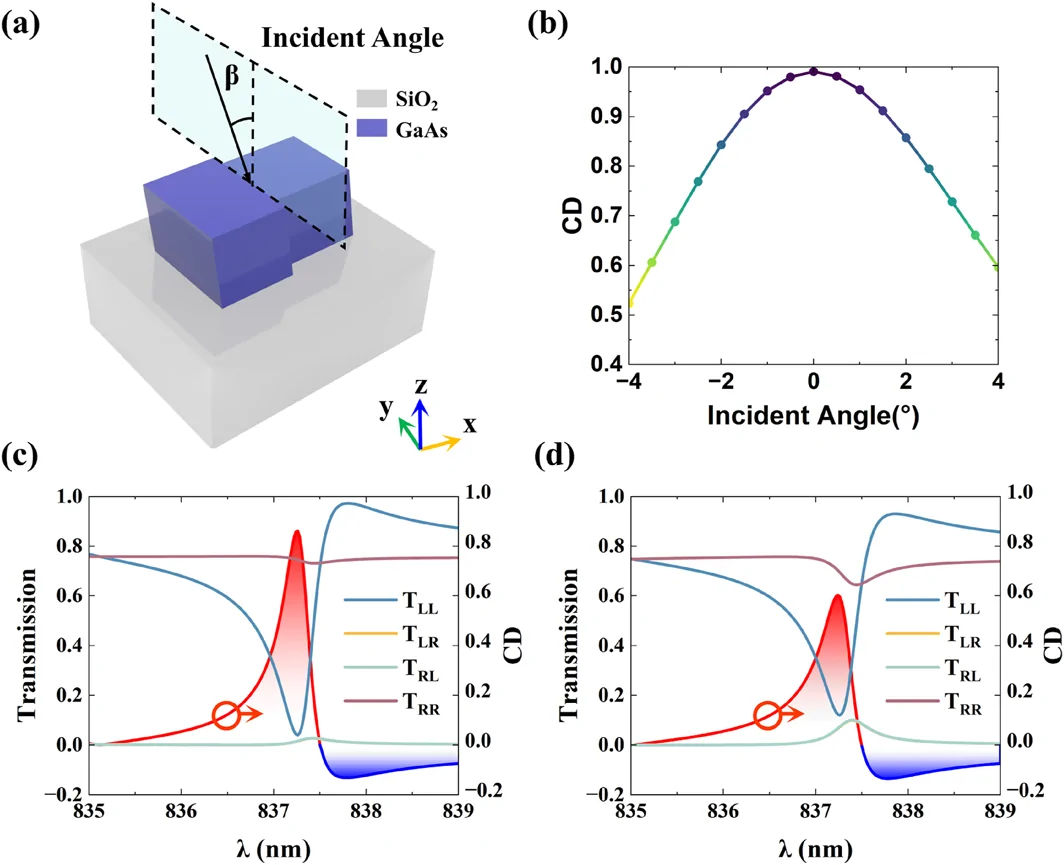
(a) Schematic of the structure with incidence at an angle *β*. (b) Simulated angle‐resolved *CD*_*T*_. The transmission spectrum and the *CD*_*T*_ spectrum of the tilted nanopillar structure at an incident angle *β* = 2° (c) and *β* = 4° (d), respectively.

Owing to the dispersionless nature of the flat band, the slow light effect provides excellent in‐plane field confinement, significantly reducing lateral leakage losses even when the mode deviates from the *Γ* point in cavities of finite sizes. As shown in the Figure 7a, we calculated the electric field distribution in a finite photonic crystal plane consisting of only eight periods. Due to the slow light effect of the flat band, the electric field is tightly localized within the lattice structure, exhibiting a quality factor of *Q*≈1108. Figure 7b shows the predicted quality factor and ellipticity for metasurfaces of varying sizes along the *y*‐direction, where *L* denotes the finite dimension of the metasurface along *y*‐direction. In a finite‐sized metasurface with dimensions of less than 4 μm, a notable quality factor of 1603 was achieved, closely approaching that of an infinitely periodic structure. Furthermore, within this finite domain, the ellipticity of the emitted radiation reached 0.99, matching the ellipticity of its infinite counterpart.

**FIGURE 7:**
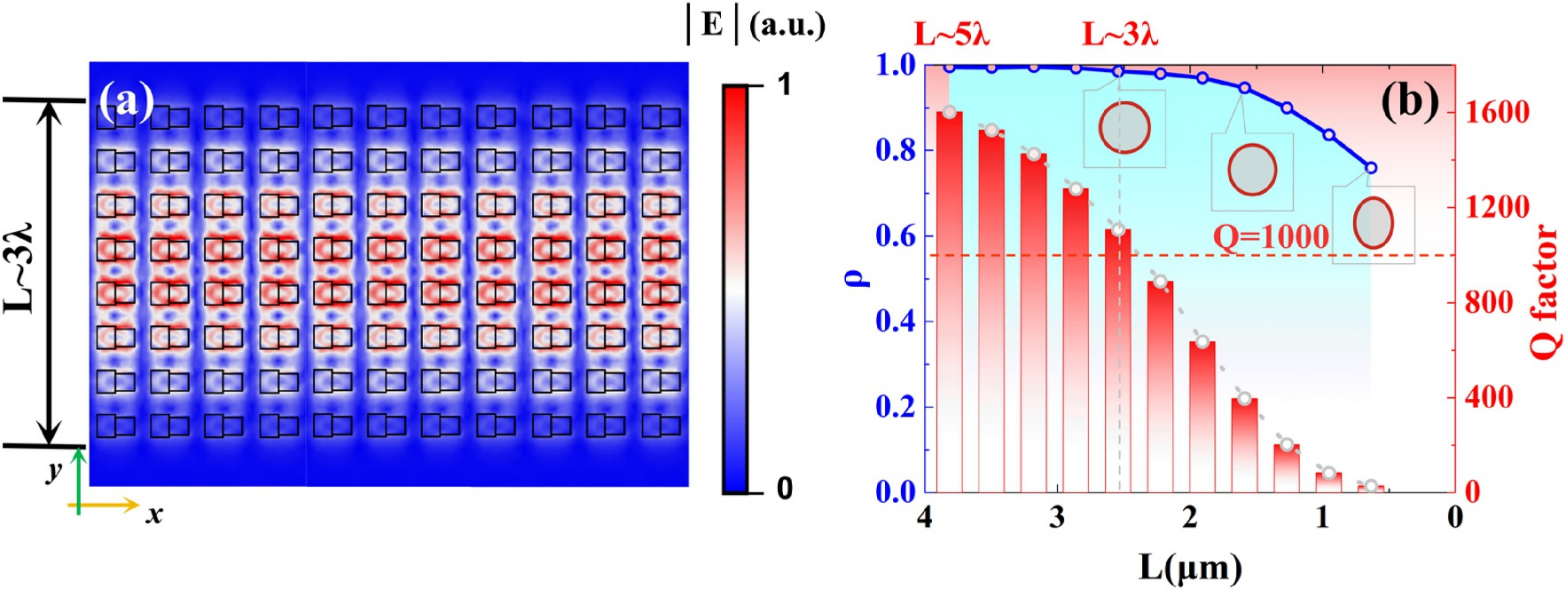
(a) Normalized electric field distribution of the semi‐infinite photonic crystal, along *x* and truncated to 8 periods along *y*. (b) Simulated quality factor and ellipticity of the structure with infinite periodicity along *y*‐direction.

Table 1 summarizes the theoretical Q factor and CD data from representative studies on chiral and/or flat‐band metasurfaces. Although structures exhibiting either flat‐band dispersion or chiral responses have been extensively studied, successfully marrying flat‐band physics with chiral geometries remains a significant challenge. Here, we demonstrate a chiral structure possessing flat‐band characteristics that achieves high CD. Our design advances the state of the art, exhibiting superior performance in both the Q factor and CD compared to previous studies, while maintaining stable resonance and high transmission circular dichroism over a moderate angular range. Furthermore, our proposed structure maintains excellent performance even with finite dimensions, which holds promising potential for application in low‐threshold, highly compact chiral lasers. A systematic tolerance analysis (Supporting Information S1) further confirms the robustness of the design against typical fabrication imperfections, supporting its feasibility for experimental realization. It offers a novel pathway toward developing energy‐efficient, monolithically integrated, and ultracompact chiral light sources.

**TABLE 1:** Comparison of the flat‐band chiral characteristics with literature.

Ref	Q factor	Finite size	Circular dichroism	Band type	Angular robustness
[19]	∼2663	200 μm	∼1	Dispersive	/
[32]	10^5^	/	None	Dispersive	± 10°
[25]	∼2840	∼3λ	None	Flat	/
[27]	∼1816	/	None	Flat	/
[33]	< 300	/	None	Flat	/
[29]	< 400	/	∼0.6	Flat	± 5°
This work	∼1108	∼3λ in *y*‐direction	∼1	Flat	± 4°

*Note:* The symbol “/” indicates that the result is not reported in the original work.

## 3 Conclusion

In summary, we demonstrate a flat‐band chiral metasurface enabled by tilted double rectangular nanopillars that break both in‐plane and out‐of‐plane mirror symmetries. This architecture simultaneously yields an ultrahigh‐Q resonance (*Q* ≈ 1969), originating from perturbed symmetry‐protected BICs and giant intrinsic transmission circular dichroism (*CD*_*T*_ ≈ 1). By engineering lattice periodicity and interpillar coupling, we collapse the photonic band into an ultraflat profile, freezing both resonance frequency and *CD*_*T*_ across moderate angular excursions. The inherent slow light effect provides exceptional in‐plane field confinement, suppressing lateral leakage and size‐induced radiative losses. Consequently, highly elliptically polarized light (*ρ* > 0.99) persists even in finite lattice of limited dimensions (as compact as ∼ 3λ wide). This platform provides an ideal option for developing compact, highly integrated circularly polarized light sources, suitable for realizing ultralow‐threshold and highly integrated chiral PCSELs. Our work concurrently achieves high‐performance chiral flat‐band operation and establishes a scalable design paradigm that synergistically integrates symmetry breaking and band flattening engineering, offering both theoretical framework and structural bases for next‐generation–integrated chiral photonic chips.

## Author Contributions

**Chunhao Xu:** conceptualization, methodology, software, data curation, formal analysis, validation, investigation, writing – original draft. **Guofeng Song:** writing – review and editing, funding acquisition, visualization, project administration, resources, supervision.

## Funding

The Strategic Priority Research Program of the Chinese Academy of Sciences (XDB0980000). Fundamental and Interdisciplinary Disciplines Breakthrough Plan of the Ministry of Education of China (JYB2025XDXM121).

## Conflicts of Interest

The authors declare no conflicts of interest.

## Supporting information


Supporting Information S1

## Data Availability

The data that support the findings of this study are available from the corresponding author upon reasonable request.
