# Supplementary material for: Giant Circular Dichroism in Chiral Broad Flat‐Band Quasi‐BIC Metasurface
Source: Nanophotonics. 2026 Apr 25;15(9):e70106. doi: 10.1002/nap2.70106 (PMC13642472; doi:10.1002/nap2.70106)
Supplement: Supplementary file 1 [file NAP2-15-e70106-s001.docx]

**Giant circular dichroism in chiral broad flatband quasi-BIC metasurface**

*Chunhao Xu,^1,3^ Jietao Liu,^2,*^ and Guofeng Song^1,3,**^*

*1 Institute of Semiconductors, Chinese Academy of Sciences, Beijing 100083, China.*

*2 Institute of Intelligent Photonics, Nankai University, Tianjin, 300071, China.*

*3 College of Materials Science and Opto-Electronic Technology, University of Chinese Academy of Sciences, Beijing 100049, China.*

** liujietao@nankai.edu.cn*

*** sgf@red.semi.ac.cn*

**A: Calculation of ellipticity**

**B: Calculation of CD**

**C: Systematic tolerance analysis**

**A: Calculation of ellipticity**

By calculating the electric field size *E_x_*(x,y) and *E_y_*(x,y) of the output beam at z = 2500 nm, the complex radiation field *r_x_*, and *r_y_* above the PC can be determined by:

$$r_{x}=\frac{1}{S}\text{∬}_{S}^{ } E_{x}\left(x,y\right)e^{-i\cdot(k_{x}\cdot x+k_{y}\cdot y)}dxdy$$

$$r_{y}=\frac{1}{S}\text{∬}_{S}^{ } E_{y}\left(x,y\right)e^{-i\cdot(k_{x}\cdot x+k_{y}\cdot y)}dxdy$$

where *r_x_* and *r_y_* are the complex radiation fields above the PC surface, *k_x_* and *k_y_* are the wave vectors, *S* is the area of a lattice unit, and z = 0 is defined in the upper layer of the PC layer. The Ellipticity can be expressed as:

$$\rho=\frac{\left|r_{x}+ir_{y}\right|-\left|r_{x}-ir_{y}\right|}{\left|r_{x}+ir_{y}\right|+\left|r_{x}-ir_{y}\right|}$$

**B: Calculation of CD**

$$T_{L}=T_{LR}+T_{LL}$$

$$T_{R}=T_{RR}+T_{RL}$$

where *T_L_* and *T_R_* are the transmittance of left-handed and right-handed circularly polarized light. The *CD_T_* can be expressed as:

$$CD_{T}=\frac{T_{L}-T_{R}}{T_{L}+T_{R}}$$

**C: Systematic tolerance analysis**

To evaluate the robustness of the proposed chiral flat-band metasurface against inevitable fabrication imperfections, we performed a systematic tolerance analysis. The key geometric parameters that critically influence the device performance—namely, the nanopillar thickness *t*, the tilt angle *θ*, and the in-plane asymmetry parameter Δ*b*—were varied within ranges representative of typical nanofabrication uncertainties. The band flatness, quality factor Q, ellipticity ρ and circular dichroism CD were calculated for each parameter set using full-wave finite-element simulations (COMSOL Multiphysics). The results are summarized in **Figure S1**.

**Figure S1.** Tolerance analysis summary: simulated band flatness, quality factor Q, ellipticity *ρ* and circular dichroism CD under variations in thickness *t*, tilt angle *θ*, and in-plane asymmetry Δ*b*.

The analysis confirms that, under reasonable parameter variations, the structure maintains high band flatness (Δλ/λ < 0.0005), high ellipticity (ρ > 0.7), high Q-factor (>1500), and high circular dichroism (CD > 0.9), supporting its feasibility for experimental realization.
